# Ventilation distribution assessed by electrical impedance tomography during robot-assisted laparoscopic prostatectomy: A randomized controlled trial

**DOI:** 10.1097/MD.0000000000051415

**Published:** 2026-10-30

**Authors:** Masaaki Michida, Yuichiro Toda, Ichiro Ohashi, Tatsuya Yamamoto, Naoto Ikemoto, Katsuhisa Kawaguchi, Yukiko Yoshida, Hajime Hinenoya, Masao Hayashi, Yoko Ochiai, Hiroshi Katayama

**Affiliations:** a Department of Anesthesiology and Intensive Care Medicine, Kawasaki Medical School, Okayama, Japan.

**Keywords:** center of ventilation, electrical impedance tomography, pneumoperitoneum, positive end-expiratory pressure, robot-assisted laparoscopic prostatectomy, Trendelenburg position

## Abstract

**Background:**

During robot-assisted laparoscopic prostatectomy (RALP), the combination of intra-abdominal pressure and a steep Trendelenburg position increases the risk of alveolar collapse and shifts the center of ventilation toward the abdomen, leading to intraoperative and postoperative hypoxia. This study evaluated whether fixed positive end-expiratory pressure(PEEP) 10 cmH_2_O, compared with zero PEEP (ZEEP), maintained a more central ventilation distribution during RALP as assessed by electrical impedance tomography (EIT).

**Methods:**

Sixty-five adult patients undergoing RALP were randomly assigned to either a PEEP 0 cmH_2_O group or a PEEP 10 cmH_2_O group. The primary endpoint was the mean center of ventilation (COV), as assessed using EIT. Secondary endpoints included other EIT parameters: the region of interest, global inhomogeneity index, and regional ventilation delay, as well as the P/F ratio, pulmonary compliance, peak airway pressure, and driving pressure (ΔP). Hemodynamic effects, lung injury biomarkers, and inflammatory cytokines were also evaluated. EIT and ventilator parameters were recorded at 6 time points: pre-intubation, post-intubation, during the Trendelenburg position, during pneumoperitoneum, pre-extubation, and post-extubation. Arterial blood gas and hemodynamic data were collected at the same points, plus 4 hours after ICU admission and again at ICU discharge. Lung injury biomarkers and inflammatory cytokines were measured pre- and postoperatively.

**Results:**

The group-by-time interaction for COV was not statistically significant (*P* = .057). In exploratory Tukey-adjusted comparisons, COV was closer to 50% in the PEEP 10 group after intubation (PEEP 0 vs PEEP 10: 58.2% vs 53.4%; *P* = .038) and before extubation (58.6% vs 53.1%; *P* = .007). ΔP was lower, and lung compliance was higher, in the PEEP 10 group during the Trendelenburg position and pneumoperitoneum. No improvement in the P/F ratio or consistent between-group differences in major hemodynamic variables, KL-6, or IL-6 were observed.

**Conclusion:**

During RALP, with a fixed PEEP of 10 cmH_2_O, there was no group-by-time interaction effect observed for COV. However, in exploratory time-point comparisons, it was associated with a tendency for ventilation to be more centrically shifted after intubation and before extubation, and was also associated with an attenuated increase in ΔP and an attenuated decrease in lung compliance during the Trendelenburg position and pneumoperitoneum.

## 1. Introduction

Robot-assisted laparoscopic radical prostatectomy (RALP) requires pneumoperitoneum and a steep head-down (Trendelenburg) position, which can lead to persistent atelectasis and alveolar damage, thereby potentially increasing the risk of perioperative respiratory complication (PPC) during and after surgery.^[1]^

During general anesthesia, it is well known that alveolar collapse can lead to a reduction in functional residual capacity and an increase in intrapulmonary shunting, both of which negatively affect oxygenation.^[2]^ To prevent these issues, the addition of positive end-expiratory pressure (PEEP) during mechanical ventilation in the intensive care unit (ICU) is common practice, and its use during general anesthesia has also been reported to be effective.^[3]^ However, a large observational study found that PEEP was not used in 40% of cases under general anesthesia.^[4]^ Although there have been reports that individualized PEEP improves ventilation during RALP with a steep head-down position and pneumoperitoneum, the primary outcome in these studies was improvement in intraoperative oxygenation as assessed by blood gas analysis.^[5]^

Electrical impedance tomography (EIT) is a technique that noninvasively visualizes changes in lung gas volume, that is, ventilation distribution, as dynamic images. EIT is used for mechanical ventilation management in critically ill patients and during surgery, particularly for determining appropriate PEEP levels.^[6,7]^

The center of ventilation (COV), measured by electrical impedance tomography (EIT), quantifies the distribution of tidal ventilation along the ventral–dorsal axis. In this study, a COV value closer to 50% was interpreted as a ventilation distribution located closer to the center of the thorax.

Patients receiving mechanical ventilation in the ICU are known to experience a shift of the center of ventilation (COV) towards the abdominal side compared to normal, due to the effects of the supine position and the development of atelectasis or pleural effusion in the dorsal region. This shift necessitates higher driving pressure (ΔP) to achieve adequate tidal volumes and may lead to decreased lung compliance. Similar phenomena are believed to occur under general anesthesia, where the supine position and muscle relaxants are used. However, no studies have quantitatively evaluated the extent to which the addition of PEEP improves the shift of the COV towards the abdominal side during RALP using the COV parameter.

Our primary hypothesis was that PEEP 10 cmH_2_O would maintain COV closer to 50% than ZEEP (0 cmH_2_O), indicating a more central ventilation distribution.

We chose a fixed PEEP of 10 cmH_2_O rather than individualized PEEP because individualized titration requires recruitment maneuvers and incremental adjustments, which prolong anesthesia and carry potential hemodynamic risks. A fixed level is more feasible in routine practice, and ZEEP (0 cmH_2_O) remains in use in many operating rooms, allowing us to maximize physiological contrast.

In our preliminary study (ethics approval no. 5137-00), when a fixed PEEP of 5 cmH_2_O was applied during RALP, we observed decreased lung compliance and increased peak airway pressure (Ppeak) and driving pressure (ΔP) during the Trendelenburg position and pneumoperitoneum. These changes did not fully recover even immediately before extubation. In the same preliminary analysis, we found that the center of ventilation could be appropriately assessed using COV.^[8]^ On the basis of these findings, the present study was designed to evaluate whether the addition of PEEP could appropriately maintain the center of ventilation, as measured by COV. This rationale motivated the current randomized controlled trial comparing high PEEP (10 cmH_2_O) with ZEEP.

## 2. Materials and methods

### 2.1. Study design

This study was a prospective, single-center, parallel-group, randomized controlled trial with a 1:1 allocation ratio. The framework of the trial was superiority.

This prospective, randomized controlled trial was conducted and reported in accordance with the CONSORT guidelines. The study was approved by the Ethics Committee of Kawasaki Medical School (Approval No. 5971-01), and written informed consent was obtained from all participants prior to enrollment. Ethics approval was granted on March 14, 2023. The trial was registered in the UMIN Clinical Trials Registry on February 3, 2022 (UMIN000046805), before the first participant was enrolled on July 19, 2023.

All research expenses were supported exclusively by institutional funding; no external funding was received.

### 2.2. Patients

Sixty-eight adult patients who underwent RALP at Kawasaki Medical School General Medical Center between July 19, 2023, and May 2, 2024, were randomized. Of these, 65 patients (PEEP 0, n = 31; PEEP 10, n = 34) had analyzable COV data and were included in the COV analysis.

Patients were excluded if they had severe cardiac or pulmonary disease, defined as an American Society of Anesthesiologists Physical Status (ASA-PS) classification of 4 or higher. Specific ASA-PS ≥4 exclusion criteria included: any myocardial infarction, cerebrovascular accident, or transient ischemic attack within the past 3 months; coronary artery disease with stent placement; ongoing myocardial ischemia or severe valvular disease; significantly reduced ejection fraction; sepsis; disseminated intravascular coagulation; acute kidney injury or end-stage renal disease not receiving regular dialysis.

In terms of respiratory status, patients were excluded if they had severely impaired pulmonary function, defined as a vital capacity <80% and FEV_1_% <70%, which would require additional anesthesia management. Other exclusion criteria included hemodynamic instability, an inability to fit the EIT belt to the chest, and the presence of a pacemaker.

### 2.3. General anesthesia

Anesthesia was induced with remifentanil (0.25–0.3 µg/kg/min), propofol (1.0–2.0 mg/kg), and rocuronium (0.6–0.9 mg/kg). Maintenance was with sevoflurane (1.0–2.0% end-tidal) and remifentanil (0.1–0.3 µg/kg/min). Vasopressors (phenylephrine or ephedrine) were administered as required to maintain mean arterial pressure (MAP) >65 mm Hg.

The EIT belt was positioned at the fourth intercostal space before induction of anesthesia.

The ventilator was set to volume-controlled ventilation, with tidal volume adjusted to 6 to 8 mL/kg of predicted body weight. Plateau pressure was measured using an inspiratory hold corresponding to 10% of the inspiratory phase. The inspired oxygen concentration (FiO_2_) was set to 0.4. The target peripheral capillary oxygen saturation (SpO_2_) was ≥95%, with FiO_2_ increased to >0.4 if required. The respiratory rate was adjusted to maintain end-tidal carbon dioxide (EtCO_2_) between 35 and 45 mm Hg, and the inspiratory-to-expiratory ratio was set to 1:2.

The patients were divided into 2 groups based on PEEP levels (PEEP 0 group = 0 cmH_2_O, PEEP 10 group = 10 cmH_2_O). The assigned PEEP level was communicated to the surgical team to anticipate potential effects on surgical exposure.

No standardized recruitment maneuver was performed in either group. Any transient desaturation (<92% SpO_2_) was managed by increasing FiO_2_; no patient required recruitment beyond protocol ventilation.

All ventilator settings, including the assigned PEEP levels, were applied and monitored by the attending anesthesiologist in accordance with the study protocol. No protocol deviations occurred, and all participants received the intervention as intended.

Other perioperative management, including anesthetic maintenance, ventilator settings (except for PEEP), fluid therapy, and vasopressor use, was standardized across both groups to avoid confounding.

### 2.4. Protocol

EIT was performed using the PulmoVista 500® (Dräger Medical GmbH, Lübeck, Germany). The belt used for EIT measurement was placed at the level of the 4th intercostal space before the induction of anesthesia, and measurements were taken at 6 points: pre-intubation, post-intubation, during the Trendelenburg position, during pneumoperitoneum, pre-extubation, and post-extubation. At each measurement point, EIT data quality was verified by checking electrode–skin contact and signal stability. Patients with persistent poor signal quality were excluded from COV analysis (n = 3). EIT data analysis was performed using Dräger SW EITdiag Version 1.6 (Dräger Medical GmbH, Lübeck, Germany). Because electrocautery caused artifacts, a short, stable recording segment was selected for analysis during pneumoperitoneum. Arterial blood gas and FloTrac data were collected at the same 6 time points as well as at 2 additional time points: 4 hours after ICU admission and at ICU discharge. No therapeutic interventions were made based on these measurements.

During surgery, vital signs (heart rate [HR], blood pressure, and SpO_2_), ventilator parameters (peak airway pressure and plateau pressure), total fluid volume, and blood gas data (pH, PaO_2_, and PaCO_2_) were recorded. The Trendelenburg position angle was approximately 23 degrees. The intra-abdominal pressure during pneumoperitoneum was initially set to 12 mm Hg in the supine position and lowered to 8 mm Hg while in the Trendelenburg position.

Patient background was assessed preoperatively through medical records or a questionnaire: age, sex, height, weight, body mass index, smoking history, alcohol consumption, medical history, comorbidities, medication history, laboratory tests (complete blood count, biochemistry, coagulation), pulmonary function tests, cardiac function tests, Sequential Organ Failure Assessment (SOFA) score, Assess Respiratory Risk in Surgical Patients in Catalonia (ARISCAT) score, and ASA-PS.

The timing for measuring KL-6 and IL-6 was at anesthesia induction, with blood gas sampling from the arterial line, and during the postoperative blood draw in the ICU the following morning.

### 2.5. Primary outcome measure

The primary outcome was the mean center of ventilation (COV) across all time points. COV was defined as the geometric mean position of the ventilation distribution along the ventral–dorsal axis, expressed as a percentage ranging from ventral (0%) to dorsal (100%). A value closer to 50% indicated a ventilation distribution located nearer the center of the thorax. The primary hypothesis was that a PEEP of 10 cmH_2_O would maintain the COV closer to 50% than zero end-expiratory pressure (ZEEP) during RALP.

### 2.6. Secondary outcome measures

Supplementary measures were region of interest (ROI), global inhomogeneity index (GI index), regional ventilation delay (RVD), P/F ratio, pulmonary compliance, peak airway pressure, and ΔP.

Additional outcome measures used to assess various aspects of patient status included background characteristics; hemodynamic variables such as HR, mean arterial pressure, cardiac index (CI), cardiac output (CO), and stroke volume variation (SVV); and arterial blood gas values. Serum levels of Krebs von den Lungen-6 (KL-6) and interleukin-6 (IL-6) were measured as markers of lung injury.

KL-6 and IL-6 were selected because KL-6 reflects type II alveolar epithelial cell injury, and IL-6 is a proinflammatory cytokine associated with perioperative pulmonary inflammation and postoperative respiratory muscle weakness.^[9]^

### 2.7. Planned sample size and rationale

The sample size was calculated using an online tool (http://powerandsamplesize.com/Calculators/). Based on preliminary experimental data, the lowest and highest mean values of the center of ventilation (COV) were 52% and 64%, respectively. Assuming a COV of 64% for the PEEP 0 group and 52% for the PEEP 10 group, with a standard deviation of 18 (based on variation in the preliminary study), and setting the type I error (α) at 0.05 and type II error (β) at 0.2 (i.e., power of 80%), the required sample size was calculated to be 36 patients per group. To account for potential dropouts, we planned to enroll 40 patients per group, resulting in a total target sample size of 80 patients. After 68 patients had been randomized, we conducted the first and only analysis of the primary outcome performed during the enrollment period using data from the 65 patients with analyzable COV data. Before this analysis, no outcome data had been analyzed, and no between-group comparisons had been performed. Based on the results of this analysis, further enrollment was discontinued before the planned sample size of 80 patients was reached.

### 2.8. Randomization and masking

Participants were randomly assigned in a 1:1 ratio to either the PEEP 0 or PEEP 10 group using computer-generated block randomization. The allocation sequence was generated with a fixed block size of 4 using an online randomization tool (https://www.sealedenvelope.com/simple-randomiser/v1/). No stratification was used. Participant enrollment and assignment were performed by the author (MM). The allocation sequence was concealed within the web-based tool and was revealed only after each participant had been registered, which prevented the investigator from foreseeing or manipulating group allocation.

Participants were blinded to group allocation because the intervention (PEEP setting) was administered under general anesthesia. Because the intervention was delivered while patients were unconscious and involved no observable or sensory differences for participants, participant blinding was considered adequate. Care providers and investigators were not blinded because of the nature of intraoperative ventilatory management.

### 2.9. Statistical analysis

Statistical analyses were performed using JMP Student Edition version 19.1.4 (JMP Statistical Discovery LLC, Cary). Patient characteristics were compared using the unpaired Student *t* test for continuous variables and Fisher’s exact test for categorical variables. Continuous variables are presented as means (95% confidence intervals), and categorical variables as n (%). COV and repeatedly measured secondary outcomes were analyzed using linear mixed-effects models with group, time point, and the group-by-time interaction as fixed effects and patient as a random effect. For COV, the group-by-time interaction was used as the primary overall test. Values for each group at each time point are presented as means estimated from the linear mixed-effects models with 95% confidence intervals. Multiple comparisons among group-by-time combinations were adjusted using the Tukey HSD method, and the tables present comparisons between the PEEP 0 and PEEP 10 groups at each time point. Time-point-specific between-group comparisons of COV and analyses of secondary outcomes were interpreted as exploratory. All tests were 2-sided, and *P* < .05 was considered statistically significant.

### 2.10. Assessment of adverse events

Potential adverse effects related to mechanical ventilation and PEEP application were systematically assessed. Hemodynamic parameters (e.g., MAP, SVV) were recorded at predefined time points throughout the perioperative period. Serum levels of Krebs von den Lungen-6 (KL-6) and interleukin-6 (IL-6) were measured preoperatively and on postoperative day 1 to evaluate potential lung injury. Skin complications related to the use of the EIT belt were assessed immediately after surgery, at the time of belt removal. No additional procedures were performed solely for the purpose of adverse event detection.

### 2.11. Patient and public involvement

Patients and members of the public were not involved in the design, conduct, or reporting of this study.

## 3. Results

### 3.1. Study population

A total of 68 patients were enrolled. Of these, 33 were allocated to the PEEP 0 group and 35 to the PEEP 10 group. Due to poor EIT signal quality, the primary outcome could ultimately only be analyzed for 31 patients in the PEEP 0 group and 34 patients in the PEEP 10 group (Fig. 1). Patient characteristics are shown in Table 1. There were no significant differences between the groups in physical characteristics (age, height, weight), preoperative variables (including ASA-PS), or intraoperative factors (surgical duration and volume of fluid administered). The SOFA score was significantly lower in the PEEP 10 group (PEEP 0 vs PEEP 10: 0.48 vs 0.18; 95% confidence interval: 0.28–0.68 vs 0.02–0.37; *P* = 0.035), though both groups had overall low scores. The number of patients who received intraoperative vasopressors was 10 in the PEEP 0 group and 12 in the PEEP 10 group (*P* = 0.80). There were also no significant differences in the mean doses of ephedrine (1.2 vs 2.4 mg, *P* = 0.19) or phenylephrine (0.02 vs 0.01 mg, *P* = 0.12).

**Table 1:** Patient characteristics and perioperative data.

	PEEP 0 (n = 33)	PEEP 10 (n = 35)	*P* value
Age (yr)	70.9 (68.3–73.4)	69.2 (66.8–71.7)	.35
Height (cm)	166.9 (164.3–169.5)	167.7 (165.2–170.2)	.63
Weight (kg)	64.9 (60.8–68.9)	68.6 (64.7–72.5)	.20
BMI (kg/m^2^)	22.6 (20.8–24.4)	23.6 (21.9–25.3)	.41
ASA physical status			.67
I	4 (12.9%)	3 (8.8%)	
II	25 (80.7%)	30 (88.2%)	
III	2 (6.5%)	1 (2.9%)	
ARISCAT score	24.2 (22.5–25.9)	23.6 (21.9–25.3)	.60
SOFA score	0.48 (0.28–0.68)	0.18 (0.02–0.37)	.04
Hypertension	15 (48.4%)	9 (26.5%)	.07
Diabetes	6 (19.4%)	2 (5.9%)	.06
Heart disease	3 (9.7%)	2 (5.9%)	.57
Respiratory disease	6 (19.4%)	5 (14.7%)	.62
Smoking			.78
Ex-smoker	13 (41.9%)	17 (50.0%)	
Current smoker	6 (19.4%)	5 (14.7%)	
Alcohol	21 (67.7%)	22 (64.7%)	.80
Duration of surgery (min)	184.0 (170.2–197.8)	188.8 (175.6–202.0)	.62
Intraoperative fluid (mL)	1684.8 (1523.7–1845.9)	1751.3 (1597.5–1905.1)	.55
Blood loss + urine output (mL)	280.5 (190.7–370.4)	340.7 (254.9–426.5)	.34
Intraoperative vasopressor	10 (32.3%)	12 (35.3%)	.80
Ephedrine (mg)	1.2 (−0.2–2.5)	2.4 (1.1–3.6)	.19
Phenylephrine (mg)	0.02 (0.01–0.04)	0.01 (0.01–0.02)	.12

Data are presented as mean (95% confidence interval) for continuous variables and n (%) for categorical variables.

*P* values represent comparisons between the PEEP 0 and PEEP 10 groups.

Ephedrine and phenylephrine doses were calculated for all patients, with a dose of zero assigned to patients who did not receive the drug.

ARISCAT = Assess Respiratory Risk in Surgical Patients in Catalonia, ASA = American Society of Anesthesiologists, BMI = body mass index, PEEP = positive end-expiratory pressure, SOFA = Sequential Organ Failure Assessment.

**Figure 1.**
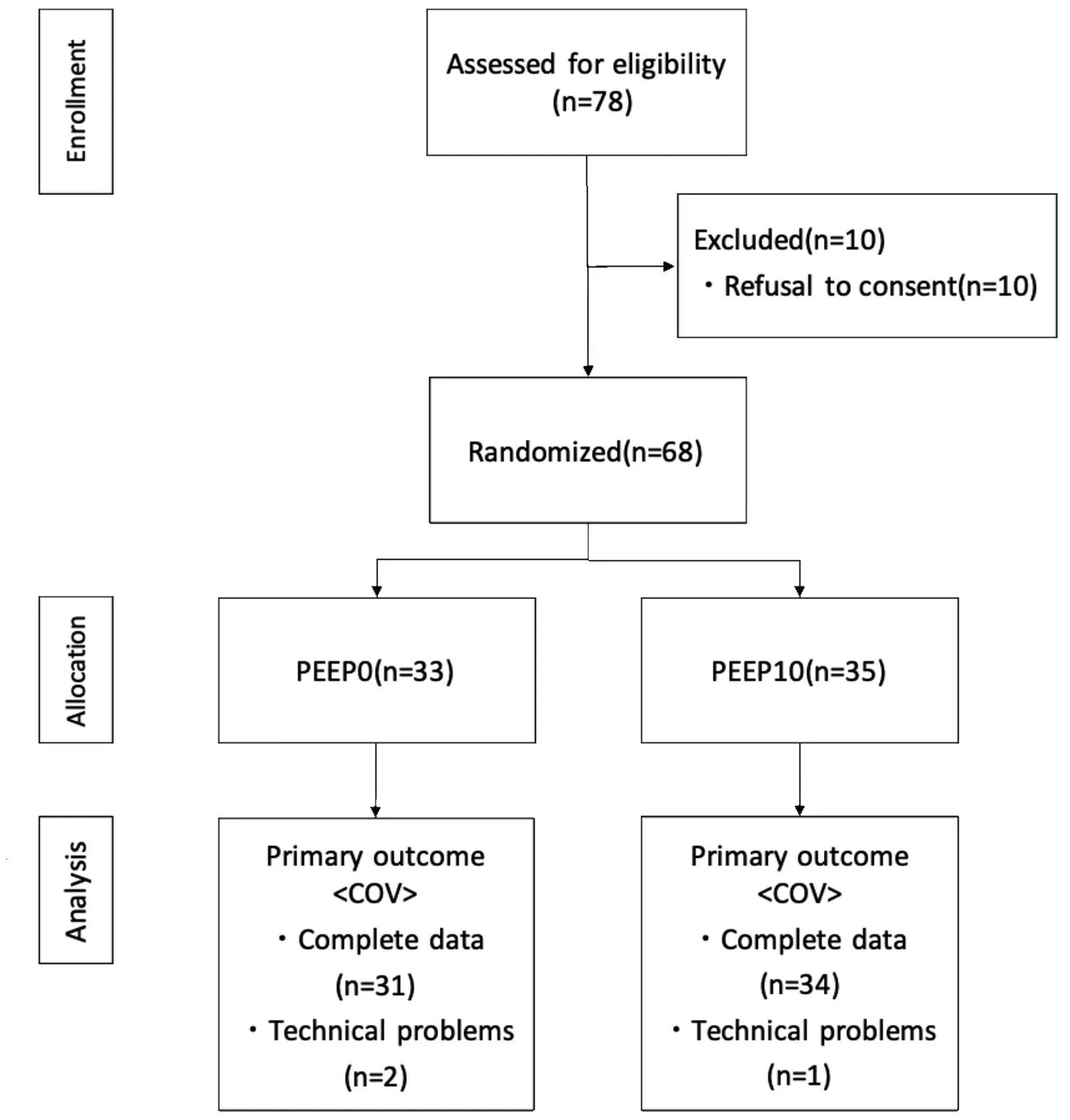
Study flowchart. COV = center of ventilation, PEEP = positive end-expiratory pressure.

### 3.2. Primary outcome: COV

The group-by-time interaction for COV was not statistically significant (*P* for interaction = 0.057; Fig. 2), providing insufficient evidence that changes in COV over time differed between the groups (Table 2). However, both group and time point were significant main effects (Table 3). In exploratory Tukey-adjusted comparisons at individual time points, post-intubation COV was 58.2% (95% confidence interval, 56.5–59.9) in the PEEP 0 group and 53.4% (51.8–55.0) in the PEEP 10 group, and was closer to 50% in the PEEP 10 group (adjusted *P* = .038). Before extubation, the corresponding values were 58.6% (56.9–60.3) and 53.1% (51.5–54.8), respectively, and the value was again closer to 50% in the PEEP 10 group (adjusted *P* = .007). The between-group differences during the Trendelenburg position and pneumoperitoneum were not significant after adjustment (adjusted *P* = .20 and .14, respectively). No significant between-group differences were observed before intubation or after extubation (adjusted *P* = .97 for both).

**Table 2:** Electrical impedance tomography–derived parameters according to study group and time point.

Parameter	Time point	PEEP 0, LS mean (95% CI)	PEEP 10, LS mean (95% CI)	Adjusted P*	*P* for interaction
ROI 1 (%)	Pre-intubation	11.1 (9.6–12.6)	9.6 (8.1–10.9)	>.99	.03
	Post-intubation	16.5 (14.6–18.4)	12.6 (10.7–14.3)	.28	
	Trendelenburg position	21.1 (18.5–23.7)	18.6 (15.7–20.7)	.88	
	Pneumoperitoneum	23.6 (20.6–26.5)	23.4 (20.6–26.1)	>.99	
	Pre-extubation	18.6 (16.5–20.7)	13.8 (11.4–15.5)	.06	
	Post-extubation	13.0 (11.4–14.5)	12.6 (11.0–14.0)	>.99	
ROI 2 (%)	Pre-intubation	40.1 (36.7–43.4)	39.4 (36.1–42.6)	>.99	.19
	Post-intubation	48.2 (44.5–51.8)	46.1 (42.7–49.6)	>.99	
	Trendelenburg position	49.0 (45.3–52.7)	49.8 (46.3–53.3)	>.99	
	Pneumoperitoneum	46.0 (42.3–49.6)	48.4 (45.0–51.8)	>.99	
	Pre-extubation	47.0 (43.7–50.3)	45.3 (42.2–48.4)	>.99	
	Post-extubation	37.6 (34.4–40.8)	38.5 (35.4–41.5)	>.99	
ROI 3 (%)	Pre-intubation	40.1 (37.0–43.2)	42.9 (40.0–45.9)	>.99	.02
	Post-intubation	29.3 (25.5–33.2)	34.7 (31.1–38.4)	.54	
	Trendelenburg position	24.0 (19.7–28.3)	25.8 (21.7–29.9)	>.99	
	Pneumoperitoneum	23.8 (20.3–27.3)	22.1 (18.8–25.3)	>.99	
	Pre-extubation	27.5 (24.2–30.9)	34.5 (31.3–37.7)	.17	
	Post-extubation	38.5 (35.4–41.5)	40.4 (37.5–43.3)	>.99	
ROI 4 (%)	Pre-intubation	6.7 (5.5–7.9)	8.2 (7.1–9.3)	.65	.89
	Post-intubation	5.9 (5.0–6.7)	6.7 (5.9–7.5)	.99	
	Trendelenburg position	5.3 (4.3–6.2)	6.1 (5.2–7.0)	>.99	
	Pneumoperitoneum	5.2 (4.3–6.2)	6.3 (5.4–7.2)	.96	
	Pre-extubation	5.6 (4.7–6.5)	6.9 (6.0–7.8)	.82	
	Post-extubation	8.1 (6.7–9.5)	8.6 (7.3–10.0)	>.99	
COV (%)	Pre-intubation	52.5 (50.9–54.0)	50.5 (49.1–52.0)	.97	.06
	Post-intubation	58.2 (56.5–59.9)	53.4 (51.8–55.0)	.04	
	Trendelenburg position	62.6 (60.0–65.1)	58.7 (56.3–61.0)	.20	
	Pneumoperitoneum	63.3 (60.6–66.0)	59.2 (56.7–61.7)	.14	
	Pre-extubation	58.6 (56.9–60.3)	53.1 (51.5–54.8)	.007	
	Post-extubation	52.5 (50.6–54.3)	50.6 (48.8–52.3)	.97	
GI index (%)	Pre-intubation	48.6 (40.6–56.7)	58.7 (51.1–66.3)	>.99	.61
	Post-intubation	49.6 (42.5–56.7)	50.8 (44.2–57.5)	>.99	
	Trendelenburg position	71.2 (45.7–96.8)	85.3 (61.3–109.3)	.95	
	Pneumoperitoneum	72.9 (61.0–84.7)	77.7 (66.8–88.6)	>.99	
	Pre-extubation	56.4 (49.8–63.0)	53.2 (47.1–59.4)	>.99	
	Post-extubation	55.1 (40.7–69.5)	65.3 (47.3–83.3)	>.99	
RVD (%)	Pre-intubation	5.4 (4.5–6.3)	6.5 (5.7–7.4)	.98	.69
	Post-intubation	4.1 (3.2–5.0)	3.9 (3.1–4.8)	>.99	
	Trendelenburg position	4.4 (3.4–5.4)	4.7 (3.8–5.7)	>.99	
	Pneumoperitoneum	5.9 (4.1–7.6)	6.7 (5.1–8.3)	>.99	
	Pre-extubation	3.5 (2.5–4.5)	3.6 (2.7–4.5)	>.99	
	Post-extubation	7.4 (5.5–9.3)	8.3 (6.5–10.1)	>.99	

Data are presented as least-squares means (95% confidence intervals).

The EIT analyses included 65 patients (PEEP 0, n = 31; PEEP 10, n = 34).

*P* values for interaction were obtained from the group-by-time interaction term in the linear mixed-effects model.

CI = confidence interval, COV = center of ventilation, GI = global inhomogeneity, LS = least-squares, PEEP = positive end-expiratory pressure, ROI = region of interest, RVD = regional ventilation delay.

* Tukey-adjusted *P* values are shown for comparisons between the PEEP 0 and PEEP 10 groups at each time point; the adjustment included all pairwise comparisons among the group-by-time least-squares means.

**Table 3:** Tests of fixed effects in the linear mixed-effects model for center of ventilation.

Effect	df (numerator, denominator)	*F* value	*P* value
Group	1, 63	11.69	.001
Time point	5, 315	70.95	<.001
Group × time point	5, 315	2.17	.06

The COV analysis included 65 patients (PEEP 0, n = 31; PEEP 10, n = 34).

The linear mixed-effects model included group, time point, and the group-by-time interaction as fixed effects and patient as a random intercept.

Degrees of freedom are presented as numerator and denominator degrees of freedom, respectively.

COV = center of ventilation, df = degrees of freedom, PEEP = positive end-expiratory pressure.

**Figure 2.**
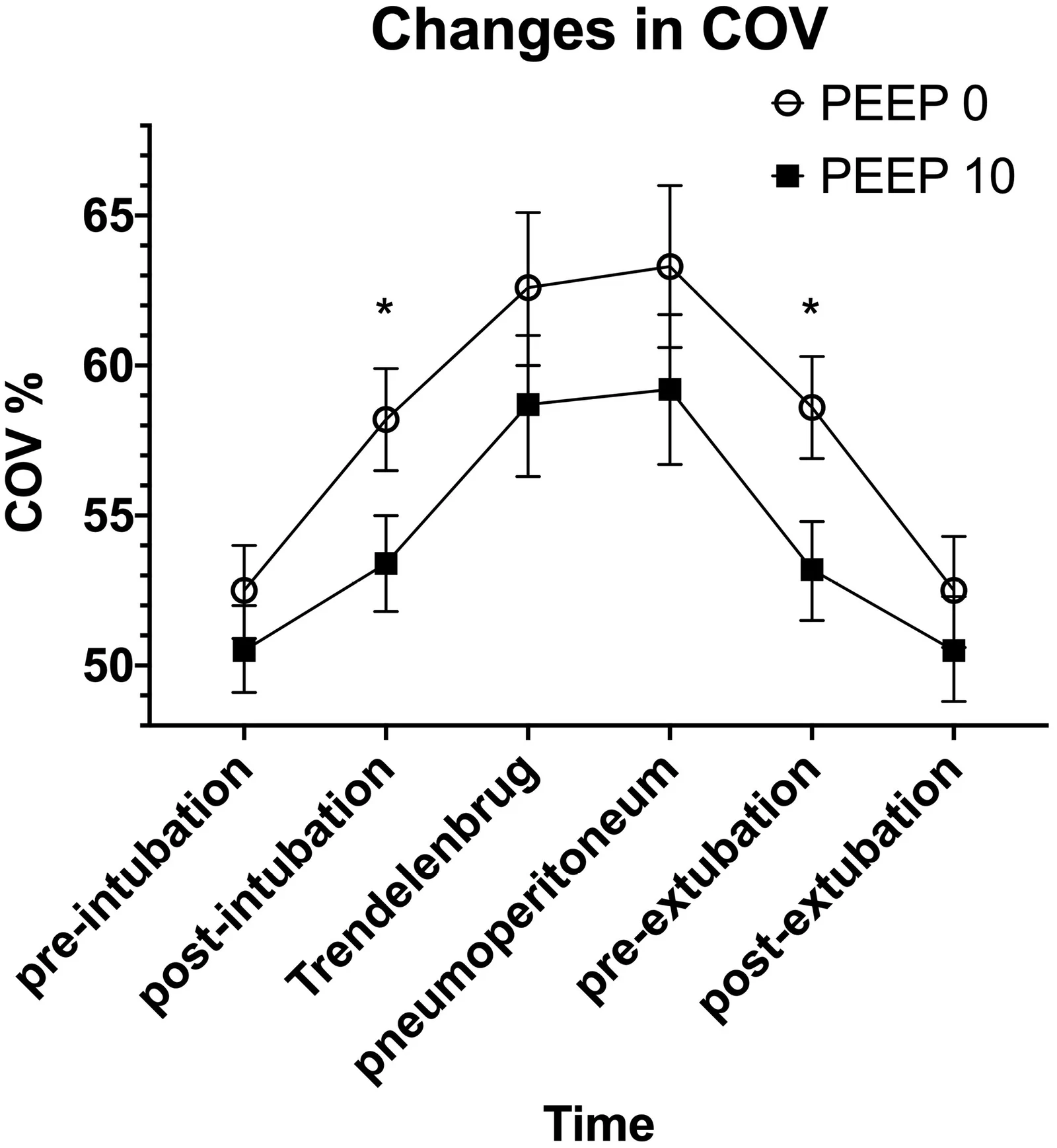
Changes in the center of ventilation. Changes in COV at each time point. White circles indicate changes in the PEEP 0 group, and black squares indicate changes in the PEEP 10 group. COV = center of ventilation, PEEP = positive end-expiratory pressure.

### 3.3. Secondary outcomes

#### 3.3.1. Respiratory outcomes

Ppeak and plateau pressure showed significant group-by-time interactions (*P* for interaction = .002 and *P* < .001, respectively). Both Ppeak and plateau pressure were higher in the PEEP 10 group at 4 time points: after intubation, during the Trendelenburg position, during pneumoperitoneum, and before extubation (Table 4). The group-by-time interaction for tidal volume was not significant (*P* = .13).

**Table 4:** Ventilatory parameters according to study group and time point.

Parameter	Time point	PEEP 0, LS mean (95% CI)	PEEP 10, LS mean (95% CI)	Adjusted P*	*P* for interaction
Peak pressure (cmH_2_O)	Post-intubation	13.5 (11.9–15.1)	19.8 (18.3–21.4)	<.001	.002
	Trendelenburg position	16.2 (15.2–17.2)	20.1 (19.1–20.9)	<.001	
	Pneumoperitoneum	20.9 (19.6–22.1)	24.1 (22.8–25.3)	.01	
	Pre-extubation	15.5 (14.2–16.8)	20.6 (19.3–21.8)	<.001	
Plateau pressure (cmH_2_O)	Post-intubation	10.9 (10.0–11.8)	16.8 (15.9–17.6)	<.001	<.001
	Trendelenburg position	13.8 (12.8–14.8)	17.7 (16.8–18.6)	<.001	
	Pneumoperitoneum	18.2 (16.9–19.6)	21.5 (20.2–22.8)	<.001	
	Pre-extubation	13.7 (12.5–14.9)	18.1 (16.9–19.2)	<.001	
PEEP (cmH_2_O)	Post-intubation	2.3 (1.6–3.0)	8.6 (7.9–9.3)	<.001	.08
	Trendelenburg position	2.2 (1.5–2.9)	8.9 (8.3–9.6)	<.001	
	Pneumoperitoneum	2.1 (1.4–2.7)	8.9 (8.3–9.5)	<.001	
	Pre-extubation	2.5 (1.8–3.1)	8.9 (8.3–9.5)	<.001	
Driving pressure (cmH_2_O)	Post-intubation	8.4 (7.8–9.0)	8.2 (7.7–8.8)	>.99	<.001
	Trendelenburg position	11.5 (10.5–12.4)	8.9 (8.0–9.7)	.005	
	Pneumoperitoneum	15.9 (14.5–17.3)	12.6 (11.3–13.8)	<.001	
	Pre-extubation	10.8 (9.8–11.7)	9.2 (8.3–10.1)	.29	
Tidal volume (mL)	Post-intubation	436.6 (410.3–462.9)	427.9 (402.7–452.9)	>.99	.13
	Trendelenburg position	427.9 (402.9–452.8)	441.0 (417.1–464.8)	>.99	
	Pneumoperitoneum	409.8 (380.3–439.2)	436.0 (407.9–464.1)	.89	
	Pre-extubation	433.2 (399.3–467.2)	450.0 (417.5–482.3)	>.99	
Lung compliance (mL/cmH_2_O)	Post-intubation	52.6 (48.7–56.5)	53.1 (49.5–56.8)	>.99	<.001
	Trendelenburg position	37.7 (34.4–41.1)	50.9 (47.8–54.1)	<.001	
	Pneumoperitoneum	26.3 (23.7–29.0)	34.4 (31.8–36.9)	.02	
	Pre-extubation	41.0 (37.1–44.8)	48.6 (44.9–52.2)	.04	

Data are presented as least-squares means (95% confidence intervals).

The ventilatory parameter analyses included 65 patients (PEEP 0, n = 31; PEEP 10, n = 34).

*P* values for interaction were obtained from the group-by-time interaction term in the linear mixed-effects model.

CI = confidence interval, LS = least-squares, PEEP = positive end-expiratory pressure.

* Tukey-adjusted *P* values are shown for comparisons between the PEEP 0 and PEEP 10 groups at each time point; the adjustment included all pairwise comparisons among the group-by-time least-squares means.

Driving pressure and lung compliance showed significant group-by-time interactions (both *P* < .001). During the Trendelenburg position, driving pressure was 11.5 cmH_2_O (95% confidence interval, 10.5–12.4) in the PEEP 0 group and 8.9 cmH_2_O (8.0–9.7) in the PEEP 10 group (adjusted *P* = .005). During pneumoperitoneum, the corresponding values were 15.9 cmH_2_O (14.5–17.3) and 12.6 cmH_2_O (11.3–13.8), respectively (adjusted *P* < .001). Lung compliance was higher in the PEEP 10 group during the Trendelenburg position (adjusted *P* < .001), during pneumoperitoneum (26.3 [23.7–29.0] vs 34.4 [31.8–36.9] mL/cmH_2_O; adjusted *P* = .02), and before extubation (adjusted *P* = .04).

#### 3.3.2. Arterial blood gas

No significant group-by-time interactions were observed for pH, PaCO_2_, PaO_2_, FiO_2_, the PaO_2_/FiO_2_ ratio, hemoglobin, actual base excess (ABE), or bicarbonate (HCO_3_^−^; Table 5). For the PaO_2_/FiO_2_ ratio, *P* for interaction was.25, and no significant between-group difference was observed at any time point after Tukey adjustment.

**Table 5:** Arterial blood gas and oxygenation parameters according to study group and time point.

Parameter	Time point	PEEP 0, LS mean (95% CI)	PEEP 10, LS mean (95% CI)	Adjusted P*	*P* for interaction
pH	Pre-intubation	7.41 (7.40–7.42)	7.41 (7.40–7.42)	>.99	.09
	Post-intubation	7.40 (7.39–7.41)	7.39 (7.39–7.40)	>.99	
	Trendelenburg position	7.41 (7.40–7.42)	7.40 (7.39–7.41)	.95	
	Pneumoperitoneum	7.38 (7.37–7.39)	7.37 (7.36–7.38)	.97	
	Pre-extubation	7.39 (7.37–7.41)	7.38 (7.36–7.39)	.93	
	Post-extubation	7.35 (7.33–7.37)	7.32 (7.31–7.34)	.09	
	4 h post-extubation	7.39 (7.38–7.40)	7.38 (7.37–7.39)	>.99	
	At ICU discharge	7.41 (7.39–7.42)	7.40 (7.39–7.41)	>.99	
PaCO_2_ (mm Hg)	Pre-intubation	38.2 (37.1–39.3)	38.0 (36.9–39.0)	>.99	.45
	Post-intubation	36.5 (34.7–38.2)	37.1 (35.4–38.8)	>.99	
	Trendelenburg position	36.0 (34.6–37.3)	36.7 (35.4–38.0)	>.99	
	Pneumoperitoneum	37.7 (35.5–40.0)	40.2 (38.0–42.3)	.82	
	Pre-extubation	38.1 (36.1–40.1)	39.7 (37.8–41.6)	>.99	
	Post-extubation	43.3 (40.7–45.9)	46.1 (43.7–48.6)	.62	
	4 h post-extubation	40.2 (39.1–41.3)	40.8 (39.7–41.9)	>.99	
	At ICU discharge	39.4 (38.2–40.6)	40.5 (39.4–41.7)	>.99	
PaO_2_ (mm Hg)	Pre-intubation	92.7 (88.2–97.2)	91.3 (87.0–95.6)	>.99	.32
	Post-intubation	271.5 (248.8–294.4)	259.1 (237.3–280.9)	>.99	
	Trendelenburg position	173.6 (155.3–192.0)	189.7 (172.2–207.2)	.99	
	Pneumoperitoneum	160.3 (143.1–177.5)	174.2 (157.8–190.6)	>.99	
	Pre-extubation	175.9 (159.5–192.4)	177.4 (161.7–193.1)	>.99	
	Post-extubation	174.8 (154.9–194.6)	177.4 (158.5–196.4)	>.99	
	4 h post-extubation	131.4 (116.8–146.1)	134.8 (120.8–148.9)	>.99	
	At ICU discharge	100.2 (91.0–109.5)	90.6 (81.8–99.4)	>.99	
FiO_2_ (%)	Pre-intubation	21.0†	21.0†	>.99	.39
	Post-intubation	53.6 (50.5–56.8)	51.0 (48.0–53.9)	.92	
	Trendelenburg position	40.4 (38.2–42.5)	39.8 (37.7–41.8)	>.99	
	Pneumoperitoneum	39.4 (37.4–41.4)	39.9 (38.0–41.9)	>.99	
	Pre-extubation	43.9 (41.0–46.7)	40.8 (38.1–43.6)	.80	
	Post-extubation	42.4 (40.2–44.7)	41.6 (39.4–43.7)	>.99	
	4 h post-extubation	32.8 (31.2–34.4)	31.3 (29.8–32.8)	>.99	
	At ICU discharge	27.5 (25.7–29.3)	24.6 (23.0–26.3)	.88	
PaO_2_/FiO_2_ ratio	Pre-intubation	444.7 (426.8–462.7)	445.2 (428.0–462.3)	>.99	.25
	Post-intubation	510.3 (477.2–543.5)	521.9 (490.2–553.5)	>.99	
	Trendelenburg position	430.5 (395.8–465.2)	487.1 (455.6–518.7)	.49	
	Pneumoperitoneum	425.5 (394.9–456.0)	447.9 (418.7–477.1)	>.99	
	Pre-extubation	410.1 (376.5–443.7)	452.0 (419.9–484.0)	.91	
	Post-extubation	442.4 (394.9–489.9)	439.4 (394.0–484.7)	>.99	
	4 h post-extubation	415.0 (378.9–451.0)	436.4 (402.0–470.8)	>.99	
	At ICU discharge	371.2 (346.1–396.2)	368.4 (344.5–392.4)	>.99	
Hb (g/dL)	Pre-intubation	14.4 (13.9–14.9)	14.6 (14.2–15.1)	>.99	.89
	Post-intubation	12.8 (12.3–13.4)	13.1 (12.6–13.6)	>.99	
	Trendelenburg position	12.8 (12.3–13.3)	12.9 (12.4–13.4)	>.99	
	Pneumoperitoneum	12.5 (12.1–12.9)	12.8 (12.4–13.2)	>.99	
	Pre-extubation	11.8 (11.4–12.2)	11.9 (11.5–12.2)	>.99	
	Post-extubation	12.0 (11.6–12.5)	12.0 (11.6–12.4)	>.99	
	4 h post-extubation	13.0 (12.5–13.5)	13.0 (12.6–13.5)	>.99	
	At ICU discharge	12.7 (12.2–13.2)	12.8 (12.4–13.3)	>.99	
ABE (mmol/L)	Pre-intubation	−0.3 (−0.8 to 0.4)	−0.2 (−0.8 to 0.3)	>.99	.36
	Post-intubation	−1.9 (−2.7 to − 0.8)	−1.8 (−2.7 to − 1.0)	>.99	
	Trendelenburg position	−1.2 (−1.8 to − 0.4)	−1.6 (−2.3 to − 0.9)	>.99	
	Pneumoperitoneum	−1.8 (−2.3 to − 1.0)	−1.9 (−2.6 to − 1.3)	>.99	
	Pre-extubation	−1.8 (−2.3 to − 1.0)	−2.0 (−2.6 to − 1.4)	>.99	
	Post-extubation	−2.1 (−2.7 to − 1.2)	−2.7 (−3.4 to − 2.0)	>.99	
	4 h post-extubation	−0.7 (−1.3 to − 0.2)	−0.8 (−1.3 to − 0.3)	>.99	
	At ICU discharge	0.2 (−0.5 to 0.7)	0.6 (0.1 to 1.1)	>.99	
HCO_3_^-^ (mmol/L)	Pre-intubation	23.7 (23.1–24.4)	23.6 (23.0–24.2)	>.99	.75
	Post-intubation	22.1 (21.1–23.1)	22.2 (21.2–23.1)	>.99	
	Trendelenburg position	22.5 (21.7–23.3)	22.3 (21.6–23.1)	>.99	
	Pneumoperitoneum	22.6 (22.0–23.3)	22.6 (22.0–23.3)	>.99	
	Pre-extubation	22.5 (21.9–23.1)	22.4 (21.8–23.0)	>.99	
	Post-extubation	23.1 (22.3–24.0)	23.0 (22.1–23.8)	>.99	
	4 h post-extubation	23.6 (23.1–24.2)	23.7 (23.2–24.2)	>.99	
	At ICU discharge	24.2 (23.6–24.7)	24.8 (24.3–25.4)	>.99	

Data are presented as least-squares means (95% confidence intervals).

The arterial blood gas and oxygenation analyses included 65 patients (PEEP 0, n = 31; PEEP 10, n = 34).

*P* values for interaction were obtained from the group-by-time interaction term in the linear mixed-effects model.

ABE = actual base excess, CI = confidence interval, FiO_2_ = fraction of inspired oxygen, Hb = hemoglobin, HCO_3_^−^ = bicarbonate, ICU = intensive care unit, LS = least-squares, PaCO_2_ = arterial partial pressure of carbon dioxide, PaO_2_ = arterial partial pressure of oxygen, PEEP = positive end-expiratory pressure.

* Tukey-adjusted *P* values are shown for comparisons between the PEEP 0 and PEEP 10 groups at each time point; the adjustment included all pairwise comparisons among the group-by-time least-squares means.

† Pre-intubation FiO_2_ was fixed at 21% in all patients; therefore, 95% confidence intervals were not calculated.

#### 3.3.3. Cardiovascular outcomes

The group-by-time interaction for HR was significant (*P* for interaction = .04), but no significant between-group difference was observed at any individual time point after Tukey adjustment. MAP, CI, and CO showed no significant group-by-time interactions (*P* = .13, .76, and .49, respectively). The interaction for SVV was not significant (*P* = .06), although SVV was higher in the PEEP 10 group only after intubation (adjusted *P* = .04; Table 6).

**Table 6:** Hemodynamic parameters according to study group and time point.

Parameter	Time point	PEEP 0, LS mean (95% CI)	PEEP 10, LS mean (95% CI)	Adjusted P*	*P* for interaction
HR (/min)	Pre-intubation	72 (67–78)	69 (64–74)	>.99	.04
	Post-intubation	66 (60–71)	68 (62–73)	>.99	
	Trendelenburg position	59 (55–63)	58 (54–62)	>.99	
	Pneumoperitoneum	56 (52–59)	57 (54–60)	>.99	
	Pre-extubation	57 (54–61)	62 (58–65)	.99	
	Post-extubation	76 (71–81)	83 (78–88)	.68	
	4 h post-extubation	69 (65–73)	69 (65–73)	>.99	
	At ICU discharge	68 (64–71)	69 (65–72)	>.99	
MAP (mm Hg)	Pre-intubation	113 (107–119)	105 (100–111)	.91	.13
	Post-intubation	85 (73–97)	79 (68–91)	>.99	
	Trendelenburg position	89 (83–96)	96 (89–102)	.98	
	Pneumoperitoneum	106 (101–111)	110 (106–115)	>.99	
	Pre-extubation	87 (82–92)	85 (80–90)	>.99	
	Post-extubation	121 (117–126)	117 (113–121)	>.99	
	4 h post-extubation	94 (90–98)	87 (83–91)	.98	
	At ICU discharge	91 (86–96)	92 (88–97)	>.99	
Cardiac index (L/min/m^2^)	Pre-intubation	3.0 (2.8–3.3)	2.9 (2.7–3.2)	>.99	.76
	Post-intubation	2.4 (2.1–2.7)	2.2 (1.9–2.5)	>.99	
	Trendelenburg position	1.9 (1.7–2.1)	2.0 (1.8–2.1)	>.99	
	Pneumoperitoneum	2.2 (2.0–2.4)	2.1 (1.9–2.3)	>.99	
	Pre-extubation	2.5 (2.3–2.7)	2.3 (2.1–2.5)	>.99	
	Post-extubation	3.2 (3.0–3.5)	3.1 (2.8–3.4)	>.99	
	4 h post-extubation	3.1 (2.9–3.3)	3.2 (3.0–3.4)	>.99	
	At ICU discharge	3.0 (2.8–3.2)	2.9 (2.7–3.1)	>.99	
Cardiac output (L/min)	Pre-intubation	5.2 (4.7–5.7)	5.2 (4.7–5.7)	>.99	.49
	Post-intubation	4.1 (3.6–4.6)	3.9 (3.4–4.4)	>.99	
	Trendelenburg position	3.3 (3.0–3.6)	3.5 (3.1–3.7)	>.99	
	Pneumoperitoneum	3.8 (3.5–4.2)	3.7 (3.4–4.0)	>.99	
	Pre-extubation	4.3 (4.0–4.6)	4.1 (3.8–4.4)	>.99	
	Post-extubation	5.6 (5.1–6.1)	5.5 (5.0–6.0)	>.99	
	4 h post-extubation	5.3 (4.8–5.8)	5.9 (5.3–6.2)	.89	
	At ICU discharge	5.3 (4.7–5.7)	5.3 (4.8–5.7)	>.99	
SVV (%)	Pre-intubation	10.0 (8.3–12.2)	10.0 (8.1–12.0)	>.99	.06
	Post-intubation	10.0 (8.8–12.4)	14.0 (12.4–15.8)	.04	
	Trendelenburg position	7.0 (6.3–8.3)	8.0 (6.8–8.8)	>.99	
	Pneumoperitoneum	6.0 (5.3–7.4)	6.0 (4.8–6.8)	>.99	
	Pre-extubation	8.0 (7.2–9.4)	10.0 (8.7–10.9)	.98	
	Post-extubation	8.0 (6.8–10.1)	10.0 (8.6–11.8)	.94	
	4 h post-extubation	9.0 (7.0–10.1)	10.0 (8.3–11.3)	>.99	
	At ICU discharge	9.0 (7.5–9.8)	9.0 (7.7–9.9)	>.99	

Data are presented as least-squares means (95% confidence intervals).

The hemodynamic analyses included 65 patients (PEEP 0, n = 31; PEEP 10, n = 34).

*P* values for interaction were obtained from the group-by-time interaction term in the linear mixed-effects model.

CI = confidence interval, HR = heart rate, LS = least-squares, MAP = mean arterial pressure, PEEP = positive end-expiratory pressure, SVV = stroke volume variation.

* Tukey-adjusted *P* values are shown for comparisons between the PEEP 0 and PEEP 10 groups at each time point; the adjustment included all pairwise comparisons among the group-by-time least-squares means.

#### 3.3.4. EIT parameters

ROI 1 and ROI 3 showed significant group-by-time interactions (*P* for interaction = .03 and .02, respectively). However, no significant between-group differences were observed at any individual time point after Tukey adjustment. ROI 2, ROI 4, the global inhomogeneity (GI) index, and regional ventilation delay (RVD) showed no significant group-by-time interactions (Table 2).

#### 3.3.5. Pulmonary epithelial injury and inflammatory biomarkers

KL-6 and IL-6 were measured before anesthesia induction and on postoperative day 1, but there were no significant differences between the 2 groups (Table 7).

**Table 7:** Pulmonary epithelial injury and inflammatory biomarkers according to study group and time point.

Parameter	Time point	PEEP 0, LS mean (95% CI)	PEEP 10, LS mean (95% CI)	Adjusted P*	*P* for interaction
KL-6 (U/mL)	Preoperative	226.9 (197.2–256.7)	225.1 (196.7–253.5)	>.99	.98
	Postoperative day 1	186.8 (162.8–210.8)	184.0 (161.5–206.5)	>.99	
IL-6 (pg/mL)	Preoperative	2.4 (1.4–3.5)	2.6 (1.6–3.6)	>.99	.38
	Postoperative day 1	35.0 (28.4–41.2)	39.0 (32.7–45.2)	.60	

Data are presented as least-squares means (95% confidence intervals).

The biomarker analyses included 65 patients (PEEP 0, n = 31; PEEP 10, n = 34).

*P* values for interaction were obtained from the group-by-time interaction term in the linear mixed-effects model.

CI = confidence interval, IL-6 = interleukin-6, KL-6 = Krebs von den Lungen-6, LS = least-squares, PEEP = positive end-expiratory pressure.

* Tukey-adjusted *P* values are shown for comparisons between the PEEP 0 and PEEP 10 groups at each time point; the adjustment included all pairwise comparisons among the group-by-time least-squares means.

#### 3.3.6. Adverse events

No patient in either group developed barotrauma, severe hypoxemia, or hemodynamic instability requiring intervention. Minor hemodynamic fluctuations are detailed in Table 6.

## 4. Discussion

In this study, we compared PEEP 0 cmH_2_O (ZEEP) and PEEP 10 cmH_2_O in patients who underwent RALP under pneumoperitoneum in a steep Trendelenburg position. Regarding COV, the primary outcome of the overall analysis, the group-by-time interaction was not statistically significant. Therefore, it cannot be concluded that PEEP 10 cmH_2_O improved the temporal changes in COV throughout the perioperative period. In an exploratory adjusted comparison using Tukey’s test, COV in the PEEP 10 group was close to 50% both after endotracheal intubation and before extubation. These findings should be interpreted as exploratory and suggest that PEEP 10 cmH_2_O may be associated with a more central ventilation distribution at specific time points. Furthermore, in the Trendelenburg position and during pneumoperitoneum, the PEEP 10 group exhibited lower driving pressure and higher lung compliance. In contrast, no improvement in the PaO_2_/FiO_2_ ratio was observed.

Scaramuzzo et al reported that the “best PEEP,” calculated using EIT in obese patients during pneumoperitoneum and the Trendelenburg position, was approximately 14 cmH_2_O.^[10]^ While that study used titration to determine the best PEEP, our study employed a fixed PEEP of 10 cmH_2_O. The finding that a relatively high PEEP level was associated with an attenuated increase in ΔP is consistent with their report; however, COV was not evaluated in their study. Nestler et al also improved oxygenation using individualized PEEP in obese patients undergoing laparoscopic surgery.^[7]^ Shono et al also reported that, compared to a PEEP of 5 cmH_2_O, a PEEP of 15 cmH_2_O improved ventilation and oxygenation in the dorsal region.^[11]^ This study used a fixed PEEP level, and its evaluation criteria were similar to our findings. However, we were unable to confirm the improvement in the P/F ratio observed in Shono et al’s study. This may be due to the difference in the PEEP level used (15 cmH_2_O in Shono et al’s study vs the levels used in our study). In our study, the finding that COV approached 50% at specific time points is consistent with the physiological possibility that moderately high PEEP partially restores a ventilation distribution shifted toward the central lung. However, since the overall interaction regarding COV was not significant, this interpretation should not be generalized.

A more consistent finding in this study was the change in respiratory mechanics. The attenuated increase in driving pressure and attenuated decrease in lung compliance observed in the PEEP 10 group under Trendelenburg positioning and pneumoperitoneum possibly reflect the maintenance of end-expiratory lung volume or a reduction in alveolar collapse. Pan et al reported that individualized PEEP based on EIT (median 13 cmH_2_O) reduced driving pressure compared with a fixed PEEP of 5 cmH_2_O,^[12]^ while Buonanno et al reported that the combination of PEEP 6 cmH_2_O and recruitment maneuvers improved ventilation homogeneity and driving pressure.^[13]^ Although the intervention methods differed, our findings are consistent with these reports suggesting that higher PEEP may improve respiratory mechanics during RALP. Our study aligns with routine general anesthesia management in that we maintained a fixed PEEP of 10 and did not perform recruitment maneuvers. This approach offers the advantage of eliminating the extra work involved in titration and recruitment maneuvers during surgeries characterized by frequent intraoperative changes, such as patient repositioning and pneumoperitoneum.

In contrast, the group-by-time interaction for the PaO_2_/FiO_2_ ratio was not significant, and even after Tukey’s correction, no significant differences between groups were observed at any time point. This result differs from the improvements in oxygenation reported by Nestler et al,^[7]^ Pan et al,^[12]^ and Girrbach et al^[5]^ following the use of individualized PEEP. Possible reasons for this include the fact that our study participants were primarily nonobese, that we used a fixed PEEP value, that we did not perform recruitment maneuvers, and that we adjusted FiO_2_ according to clinical necessity. Since oxygenation is also influenced by pulmonary perfusion and ventilation-perfusion matching, changes in ventilation distribution or respiratory mechanics derived from EIT do not necessarily lead directly to an improvement in the PaO_2_/FiO_2_ ratio.

In this study, we did not perform individualized PEEP adjustments or recruitment maneuvers; instead, we used a fixed PEEP setting of 10 cmH_2_O. Because a fixed PEEP does not require additional adjustments in response to intraoperative changes such as changes in patient position or pneumoperitoneum, it is easy to implement in routine clinical practice. Although our findings suggest physiological benefits of a fixed PEEP of 10 cmH_2_O, they do not demonstrate superiority over individually adjusted PEEP or a reduction in postoperative pulmonary complications.

No clear hemodynamic deterioration associated with fixed PEEP 10 cmH_2_O was identified under the conditions of this study. However, the study was not designed to detect cardiovascular adverse events and did not evaluate potential effects of PEEP on venous return, intracranial pressure, or intraocular pressure. Therefore, no definitive conclusions regarding safety can be drawn.

## 5. Limitations

This study has several limitations.

First, no interim analysis or statistical stopping rule was prespecified. After 68 patients had been randomized, the primary outcome was analyzed for the first and only time using data from the 65 patients with analyzable COV data, and further enrollment was discontinued before the planned sample size of 80 patients was reached based on the results of this analysis. Although no analyses had been conducted before this analysis of the 65 patients, stopping enrollment on the basis of the observed results may have increased the risk of type I error and led to an overestimation of the effect size. Second, because the group-by-time interaction for COV was not statistically significant, the time-point-specific comparisons should be interpreted as exploratory. Third, interference from electrocautery limited the duration of EIT measurements, and COV could not be analyzed in 3 patients because of poor signal quality. Fourth, owing to the nature of the intervention, the anesthesiologists and investigators could not be blinded. Fifth, this was a relatively small, single-center study and was not adequately powered to evaluate clinical outcomes such as postoperative pulmonary complications. Sixth, ZEEP may not be considered a standard comparator in contemporary lung-protective ventilation. However, a multicenter observational study by Suzuki et al. reported that PEEP was not applied in approximately 40% of patients undergoing general anesthesia in Japan, indicating that ZEEP was still commonly used in clinical practice.^[4]^

## 6. Conclusion

During RALP, with a fixed PEEP of 10 cmH_2_O, there was no group × time interaction effect observed for COV. However, in exploratory time-point comparisons, it was associated with a tendency for ventilation to be more centrically shifted after endotracheal intubation and before extubation, and was also associated with an attenuated increase in driving pressure and an attenuated decrease in lung compliance during the Trendelenburg position and during pneumoperitoneum.

## Acknowledgments

The authors would like to thank all individuals who contributed to this study but are not listed as authors.

## Author contributions

**Conceptualization:** Masaaki Michida, Yuichiro Toda, Hiroshi Katayama.

**Data curation:** Masaaki Michida.

**Formal analysis:** Masaaki Michida, Yuichiro Toda.

**Investigation:** Masaaki Michida, Ichiro Ohashi, Tatsuya Yamamoto, Naoto Ikemoto, Katsuhisa Kawaguchi, Yukiko Yoshida, Hajime Hinenoya, Masao Hayashi, Yoko Ochiai, Hiroshi Katayama.

**Methodology:** Masaaki Michida, Yuichiro Toda.

**Project administration:** Masaaki Michida.

**Supervision:** Masaaki Michida.

**Validation:** Masaaki Michida.

**Visualization:** Masaaki Michida.

**Writing – original draft:** Masaaki Michida.

**Writing – review & editing:** Masaaki Michida, Yuichiro Toda.
